# How Much to Sleep

**Published:** 1876-02

**Authors:** 


### HOW MUCH TO SLEEP.

THE amount of sleep which persons require, varies with the age, habits, and conditions of men.

If we will yield to Nature's guidance, instinct will designate the exact quantity required for each, with promptitude and accuracy. All know, that a nights' full, natural sleep, gives an awaking of freshness and vigor, which insures bodily enjoyment for a whole day; but if sleep is broken and disturbed, it is certainly followed by lassitude of body and mind; this palpable fact demonstrates that body and brain, flesh and spirit, are recuperated by sleep; it then follows, that the more we work, the more we study, the more sleep we require. To ascertain how much sleep each one needs, we will give a rule presently; but it is useful to know, that Nature will not take too much sleep, except by violent and artificial means: if forced upon her long, obesity, or other form of destructive disease, is inevitable; but if we attempt to rob the body of its requisite amount, debility of body, madness of mind, or premature death, will always result, if this violence is persevered in. There are persons whose voraciousness of time is such, that they consider, that the hours spent in sleep, beyond the briefest number, are hours lost; that if they can go to bed very late, and get up very early, it is so much added to life. We once heard a man say, that no time should be lost, that a book should be always at hand, so that in waiting for dinner, or a friend, we might read, even if it were but a line. He practiced this. His was accounted one of the greatest minds in the nation; his writings will live when the names of Presidents will be repeated but once in an age. He lost his mind, and died in his prime! The truly wise will, therefore, yield themselves to Nature's apportionment. It is a law of our being, as beneficent as it is wise, that if we are let alone, we wake up of ourselves, as soon as the system has taken an amount of repose proportioned to the exertions of the previous day, and the usual ones of the day following. All that remains, therefore, for us to do, is to aid Nature in the outset, or rather avoid acting in such a way as to interfere with her operations, by simply going to bed at a regular hour, with a mind, and body, and stomach, unoppressed

